# Neuroprotection mediated by inhibition of calpain during acute viral encephalitis

**DOI:** 10.1038/srep28699

**Published:** 2016-06-27

**Authors:** Charles L. Howe, Reghann G. LaFrance-Corey, Kanish Mirchia, Brian M. Sauer, Renee M. McGovern, Joel M. Reid, Eric J. Buenz

**Affiliations:** 1 Departments of Neurology, Mayo Clinic, Rochester, Minnesota, 55905 USA; 2 Departments of Neuroscience, Mayo Clinic, Rochester, Minnesota, 55905 USA; 3 Departments of Immunology, Mayo Clinic, Rochester, Minnesota, 55905 USA; 4 Neurobiology of Disease PhD program, Mayo Graduate School, Mayo Clinic, Rochester, Minnesota, 55905 USA; 5 Division of Oncology Research, Department of Oncology, Mayo Clinic, Rochester, Minnesota, 55905 USA

## Abstract

Neurologic complications associated with viral encephalitis, including seizures and cognitive impairment, are a global health issue, especially in children. We previously showed that hippocampal injury during acute picornavirus infection in mice is associated with calpain activation and is the result of neuronal death triggered by brain-infiltrating inflammatory monocytes. We therefore hypothesized that treatment with a calpain inhibitor would protect neurons from immune-mediated bystander injury. C57BL/6J mice infected with the Daniel’s strain of Theiler’s murine encephalomyelitis virus were treated with the FDA-approved drug ritonavir using a dosing regimen that resulted in plasma concentrations within the therapeutic range for calpain inhibition. Ritonavir treatment significantly reduced calpain activity in the hippocampus, protected hippocampal neurons from death, preserved cognitive performance, and suppressed seizure escalation, even when therapy was initiated 36 hours after disease onset. Calpain inhibition by ritonavir may be a powerful tool for preserving neurons and cognitive function and preventing neural circuit dysregulation in humans with neuroinflammatory disorders.

The global burden of symptomatic viral encephalitis is approximately 1 in 10,000 people, with non-polio picornaviruses, arboviruses, and herpsesviruses accounting for the majority of cases^1^. In the US, between 1998 and 2010, almost 50,000 children under 20 years of age were hospitalized with encephalitis^2^, with subclinical encephalitis occurring in an unknown, but likely much larger number. Encephalitis during childhood results in permanent neurologic complications in up to 50% of survivors, depending upon the viral pathogen^1^, with sequelae ranging from cognitive impairment to epilepsy^3^. For example, several years after enterovirus-71 infection involving the CNS, children were more likely to exhibit inattention, impulsivity, and hyperactivity in a battery of cognitive tests^4^. Likewise, analysis of Mayo Clinic’s Olmsted County cohort over a 46 year period revealed a 16-fold increase in unprovoked seizures following viral encephalitis^5^. These findings are consistent with encephalitis-induced neuronal injury and neural circuit dysregulation.

While some neuronal loss during viral encephalitis may occur due to direct virus-mediated injury, much of the damage is associated with bystander pathology – the loss of cells that are not directly compromised by the pathogen but which are in proximity to an inflammatory focus. Neuroinflammation is generally a collaborative interaction between brain-resident cells such as microglia and astrocytes and infiltrating cells such as inflammatory monocytes, neutrophils, and other granulocytes and phagocytes. In many neurologic diseases, the complex and dynamic inflammatory response within the CNS is precariously balanced between protective and destructive outcomes. This is especially true within the context of CNS infection, in which an inflammatory response is necessary to control the pathogen but is dangerously poised to wreak havoc with the normally fine-tuned homeostasis of the brain. Damage wrought by infiltrating inflammatory cells is a direct result of the relatively untamed nature of innate immune responses. The toxic milieu created by an infiltrating inflammatory monocyte or neutrophil is well-suited to rapidly control pathogens but is ill-suited to the easily disturbed network of the CNS – a veritable bull in the china shop.

We previously showed that acute brain injury associated with infection by the Daniel’s (DA) strain of Theiler’s murine encephalomyelitis virus (TMEV) in C57BL/6J mice is the result of infiltrating inflammatory monocytes^6^,^7^. Furthermore, we have shown that hippocampal neuron death in these animals is associated with calpain activation^8^. Our working model is that infiltrating inflammatory cells release cytokines and other effector molecules that disrupt hippocampal circuitry, triggering seizures and inducing further disruption of the hippocampal network. Such a model involves both the direct killing of neurons by inflammatory responders and loss of neurons due to excitotoxicty. Based on this model, we hypothesized that intervention with a neuroprotective strategy during acute infection would preserve hippocampal circuitry and cognitive function. Moreover, based upon our analysis of the mechanisms of cell death in the acutely infected brain, we hypothesized that calpain is a prime target for therapeutic intervention.

Calpains are calcium-activated non-lysosomal cysteine proteases that cleave substrates on the basis of regional hydrophobicity and electrostatic potential interactions created by neighboring primed and unprimed position amino acids in the peptide chain of the target protein^9^. This is similar to the substrate specificity of the HIV protease, in which traditional linear amino acid sequence analyses only weakly predict the context-dependent cleavage sites^10^. The HIV protease inhibitor ritonavir was designed around a symmetric backbone that provided structured information mimicking both unprimed and primed substrate residues^11^,^12^. Of note, ritonavir also inhibits calpain, with a K_i_ of 11 μM against total cellular calpains^13^.

The present study tested the ability of ritonavir to inhibit calpain and preserve neurons in a mouse model of acute CNS picornavirus infection. Using therapeutic dosing regimens that obtained peak plasma concentrations within the range for calpain inhibition, we found that ritonavir protected hippocampal neurons, preserved cognitive function, and suppressed seizures in acutely infected mice.

## Results

### Calpain is activated in the hippocampus during acute TMEV infection

**Figure 1:**
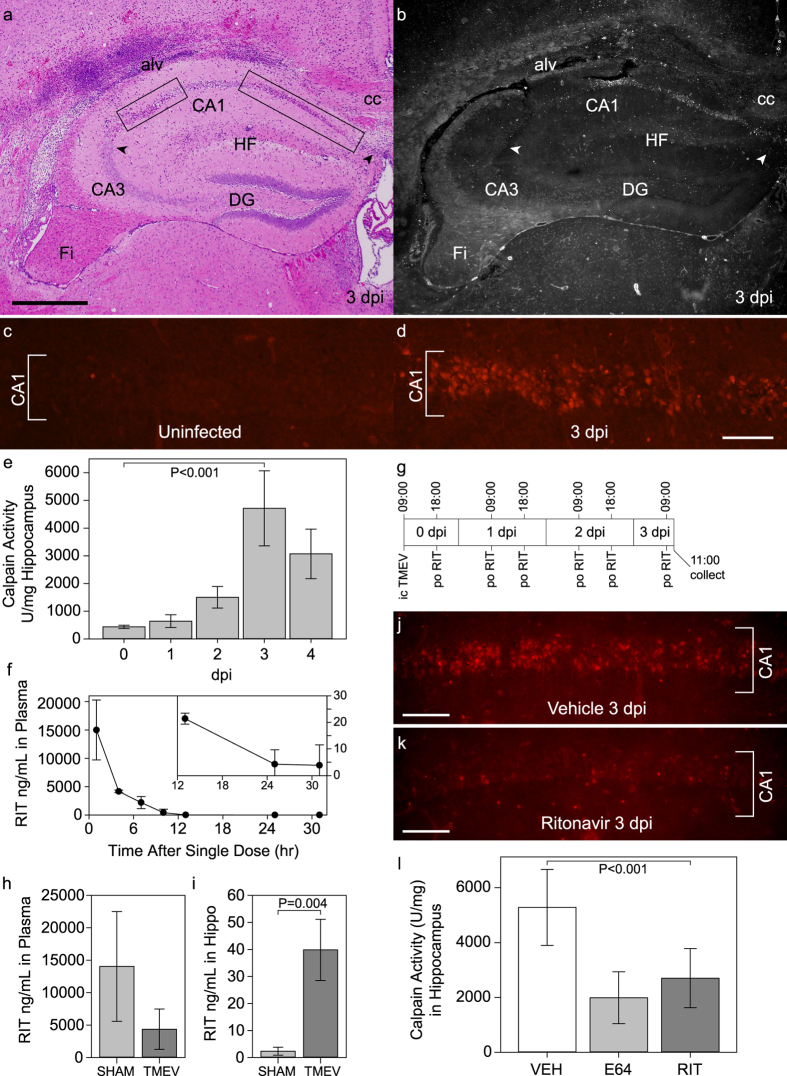
Calpain activation in CA1 hippocampal neurons during acute infection is inhibited by ritonavir treatment. (**a**) At 3 days postinfection (dpi) the CA1 hippocampal pyramidal neuron layer is marked by pyknotic nuclei (rectangular outlines); CA3 and dentate gyrus (DG) neurons are spared. Image is representative of greater than 30 individual animals. (**b**) *In situ* calpain zymography on a tissue section adjacent to (**a**) shows that calpain activity (white) is largely restricted to the CA1 layer at 3 dpi. Image is representative of 10 mice. (**c**) While no calpain activity is detected in CA1 neurons in uninfected mice, (**d**) robust calpain activity (red) is observed in CA1 neurons at 3 dpi. Images are representative of 10 mice per condition. (**e**) 3 dpi is the peak for calpain activity in the hippocampus as measured biochemically; *P *< 0.001 by one-way ANOVA; SNK pairwise analysis indicates all timepoints are different from one another, with 3 dpi vs uninfected at *P *< 0.001; n = 5 mice per timepoint. (**f**) Mice were treated by oral gavage with a single dose of the pharmaceutical formulation of ritonavir and levels of the drug in plasma were measured by HPLC over 31 hrs; n = 3 mice. (**g**) Schematic of the multiple dosing regimen used for (**h,i**). At 3 dpi ritonavir was detected in plasma (**h**) and in hippocampal lysates (**i**). Levels were elevated in the hippocampus in TMEV-infected mice as compared to sham-infected; *P* = 0.004; n = 5 mice per group. (**j**) Vehicle-treated mice showed high calpain activity in CA1 neurons at 3 dpi (compare to **d**); ritonavir robustly suppressed calpain activity in these cells (**k**). Images are representative of 5 mice per group. (**l**) Oral ritonavir was as effective as E64 at suppressing hippocampal calpain activity at 3 dpi (*P *< 0.001 between all groups by one-way ANOVA; SNK pairwise: vehicle vs RIT, *P *< 0.001; E64 vs RIT, *P* = 0.110); n = 5 mice per group. alv = alveus; cc = corpus callosum; Fi = fimbria/fornix; HF = hippocampal fissure. Scale bar in (**a)** is 500 μm and refers to (**b**) scale bar in (**d)** is 100 μm and refers to (**c**) scale bars in (**j)** and (**k)** are 100 μm. Graphs show means ± 95% confidence intervals.

Our previous studies indicated that CA1 region hippocampal neurons die by 4 days after TMEV infection in B6 mice^7^,^14^. This death was dissociated from direct virus infection of the dying neurons and was preceded by a wave of calpain activity^8^. In the present study, we observed extensive regions of neuron injury restricted to the CA1 layer in the hippocampus at 3 days postinfection (dpi) (Fig. 1a; Supplementary Fig. S1). Peak calpain activity measured in hippocampal homogenates was also observed at 3 dpi (*P *< 0.001 by one-way ANOVA) (Fig. 1e). In a separate experiment, uninfected mice and sham-infected mice at 3 dpi showed low levels of calpain activity in the hippocampus (615 ± 131 U/mg vs 621 ± 151 U/mg; *P* = 0.986 by one-way ANOVA with SNK pairwise comparison), while levels in TMEV-infected mice were 10 times higher (5695 ± 797 U/mg; *P *< 0.001 vs both uninfected and sham-infected by one-way ANOVA with SNK pairwise comparison). *In situ* calpain zymography^15^ on frozen sections of brain collected at 3 dpi revealed calpain activity that was largely restricted to the CA1 region (Fig. 1b). This activity coincided with the location of dying hippocampal neurons (Fig. 1a). While very little calpain activity was observed in the CA1 region in uninfected mice (Fig. 1c), a clear pattern of activity within CA1 pyramidal neurons was observed by 3 dpi (Fig. 1d). We conclude that by 72 hours postinfection (hpi) calpain activity is significantly increased in CA1 pyramidal neurons, a time frame that immediately precedes peak neuron death in the hippocampus^8^.

### Ritonavir treatment reduces calpain activity in the hippocampus during acute TMEV infection

Based on the high level of calpain activity observed within CA1, we hypothesized that treatment of acutely infected mice with a calpain inhibitor would protect hippocampal neurons. To determine the pharmacokinetic profile of ritonavir in mice, one dose of the pharmaceutical formulation of ritonavir (0.5 mg/g body weight) was delivered by oral gavage and levels of the drug in plasma were measured by HPLC over 31 hrs (Fig. 1f). At one hr after dosing we detected 14992 ± 5267 ng/mL circulating ritonavir, equivalent to 21 μM. The drug was still detectable in plasma at significant levels at 13 hrs after dosing (*P *< 0.001 by one-way ANOVA). As an alternative treatment regimen for some experiments we delivered ritonavir dissolved in DMSO via intraperitoneal injection (0.5 mg/g body weight) twice per day. This strategy resulted in 46 μM (33116 ± 7336 ng/mL) ritonavir in plasma at peak and 6 μM (4215 ± 1740 ng/mL) ritonavir at trough over the course of 4 days. Both delivery strategies yielded plasma levels of ritonavir that are comparable to the human pharmacokinetics (Norvir product insert: 11 μg/mL peak, 4 μg/mL trough). Using the twice daily oral gavage dosing scheme shown in Fig. 1g, with the first dose of ritonavir delivered at 9 hpi, we found that ritonavir was detected in plasma and in hippocampal homogenates in TMEV-infected mice at 3 dpi. Circulating ritonavir trended toward lower levels in TMEV-infected mice as compared to sham-infected controls (Fig. 1h) (*P* = 0.136), but levels were significantly elevated in the hippocampus (Fig. 1i) (*P* = 0.004 by t-test), suggesting that ongoing inflammation enhances access of ritonavir to the brain parenchyma. This oral dosing scheme (ritonavir at 0.5 mg/g body weight), initiated at 9 hpi, resulted in robust suppression of calpain activity in CA1 neurons at 3 dpi, as revealed by both *in situ* calpain zymography (Fig. 1j,k) and measurement of calpain activity in hippocampal lysates (Fig. 1l). Oral ritonavir was as effective as intraperitoneal delivery of the calpain inhibitor E64 (6.4 μg/g)^16^ at suppressing hippocampal calpain activity at 3 dpi (Fig. 1l). We conclude that oral ritonavir effectively reduced calpain activation in CA1 pyramidal neurons in TMEV-infected mice.

### Ritonavir protects hippocampal neurons

**Figure 2:**
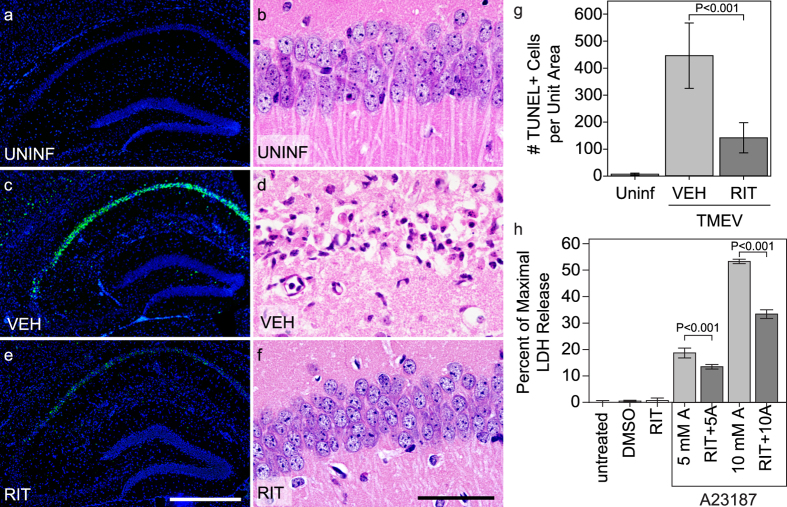
Treatment with ritonavir reduces CA1 neuron death and hippocampal injury in acutely infected mice. TUNEL staining of the hippocampal region (**a**,**c**,**e**) and histological examination of the CA1 neuron layer (**b**,**d**,**f**) in uninfected mice (**a**,**b**), 4 dpi TMEV-infected mice treated with vehicle (**c**,**d**), and 4 dpi TMEV-infected mice treated with ritonavir (**e**,**f**) showed reduced neuron cell death only in ritonavir-treated mice. (**g**) Quantitation of the number of TUNEL-positive cells revealed that ritonavir treatment significantly protected the hippocampus (*P *< 0.001 between all groups by one-way ANOVA on ranks; SNK pairwise: VEH vs RIT, *P *< 0.05; n = 10 mice per group). Area of the hippocampus analyzed was not different between the groups (*P* = 0.613 by one-way ANOVA). (**h**) Mouse hippocampal neurons were treated with the calcium ionophore A23187 (5 μM or 10 μM) for 24 hr in the presence of 10 μg/mL ritonavir or DMSO. Neuron cell death was assessed by LDH assay. Ritonavir significantly decreased cell death in response to the ionophore (*P *< 0.001 by one-way ANOVA; SNK pairwise analysis indicates *P *< 0.001 for all ritonavir vs DMSO pairs; n = 4 samples per condition). TUNEL and histology images are representative of more than 10 mice per condition. Scale bar in (**e)** is 500 μm and refers to (**a**,**c)**. Scale bar in (**f)** is 50 μm and refers to (**b**,**d)**. UNINF = uninfected; VEH = vehicle-treated; RIT = ritonavir-treated; DMSO = vehicle only; RIT = ritonavir only; 5 mM A = 5 mM A23187 + VEH; RIT + 5A = 5 mM A23187 + RIT; 10 mM A = 10 mM A23187 + VEH; RIT + 10A = 10 mM A23187 + RIT. Graphs show means ± 95% confidence intervals.

Ritonavir treatment (0.5 mg/g body weight by intraperitoneal injection every 12 hrs from time of infection to 4 dpi) also significantly reduced the number of TUNEL-positive neurons in the CA1 region of the hippocampus at 4 dpi (Fig. 2e,g) as compared to vehicle treatment (Fig. 2c,g), indicating inhibition of cell death. While over 400 TUNEL-positive neurons were counted in the CA1 layer of one standardized hippocampal field in vehicle-treated mice, fewer than 150 were detected per field in ritonavir-treated mice (vehicle vs ritonavir; *P *< 0.001) (Fig. 2g). The TUNEL-positive neurons that were observed in ritonavir-treated mice were also more spatially restricted within the CA1 layer (Fig. 2e) as compared to vehicle controls (Fig. 2c). Protection of CA1 neurons by ritonavir was confirmed by histology at 7 dpi, which routinely showed complete loss of the CA1 pyramidal cell layer in vehicle-treated mice (Fig. 2d) but nearly complete preservation of these neurons in ritonavir-treated animals (Fig. 2f). Moreover, many of the ritonavir-preserved neurons maintained apical dendrites (Fig. 2f) that resembled those observed in uninfected mice (Fig. 2b). The reduction of calpain activity and the reduction in TUNEL-positive cells within CA1 following treatment suggested that ritonavir directly protected these neurons from cell death. To test this, we incubated primary hippocampal neurons with the calcium ionophore A23187 (5 or 10 μM) for 24 hr in the presence of ritonavir (10 μg/mL) or DMSO (vehicle). A23187 triggers excitotoxic cell death that is blocked by inhibition of calpain^17^,^18^. Using a lactate dehydrogenase release assay, we found that ritonavir reduced hippocampal neuron death in response to A23187 (Fig. 2h) (P < 0.001 for ritonavir treated groups vs matched vehicle-treated groups). We conclude that ritonavir robustly protected hippocampal neurons from cell death.

### Ritonavir does not change viral biology

**Figure 3:**
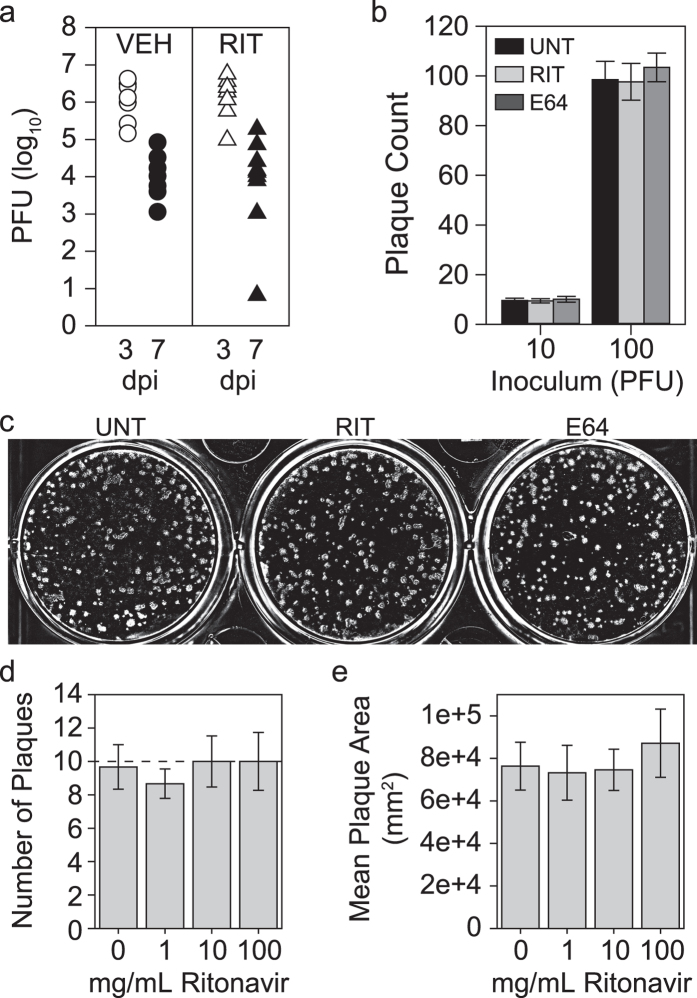
Ritonavir does not alter TMEV infection. (**a**) Viral load in the brain was assessed by plaque assay in TMEV-infected mice treated with vehicle (VEH) or ritonavir (RIT) at 3 and 7 dpi. There was no difference between treatment groups at either timepoint. n = 5 mice per treatment and per timepoint. (**b**,**c**) Ritonavir (10 μg/mL) and the standard calpain inhibitor E64 (10 μg/mL) did not alter TMEV infectivity when added directly to cells during a plaque assay. Images are representative of the n = 6 samples per condition. (**d**,**e**) Dose response analysis for ritonavir added directly to cells during TMEV infection showed no effect on infectivity (**d**; number of plaques) or viral fitness (**e**; plaque size) even at very high concentrations. n = 6 samples per condition from two independent experiments. Graphs show means ± 95% confidence intervals.

Ritonavir was originally developed as an inhibitor of the HIV protease^12^, and while the retroviral protease and the picornaviral protease share no sequence or functional homology, it is possible that ritonavir alters viral infection parameters that lead to preservation of CA1 neurons. Therefore, we measured viral load by plaque assay of brain homogenates collected from vehicle- and ritonavir-treated mice (Fig. 3a). Both groups exhibited a decrease in viral load from 3 dpi to 7 dpi, as expected, and there was no significant difference between groups at either timepoint. We verified that neither ritonavir (10 μg/mL) nor the calpain inhibitor E64 (10 μg/mL) altered virus growth when added directly to purified TMEV in the plaque assay (Fig. 3b,c). Even at a high dose of 100 μg/mL (140 μM), ritonavir had no effect on TMEV replication (Fig. 3d) or fitness (Fig. 3e). We conclude that ritonavir-mediated protection of hippocampal neurons did not involve direct effects on TMEV infectivity, replication, or kinetics.

### Ritonavir preserves cognitive function

**Figure 4:**
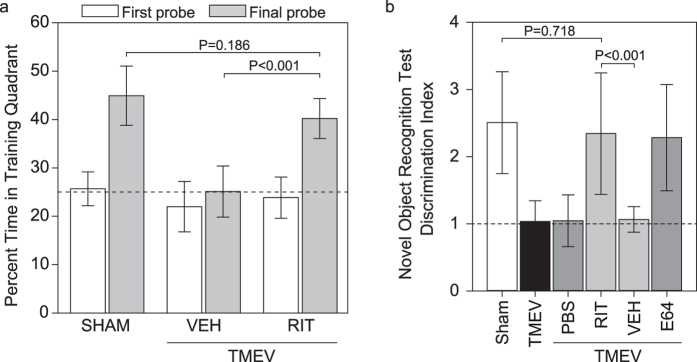
Treatment with ritonavir preserves cognitive function in acutely infected mice. Cognitive function was tested by Morris water maze (**a**) and novel object recognition (**b**) starting at 15 dpi. (**a**) The percentage of swim time spent in the training quadrant (escape quadrant) during a probe test in which the submerged platform is removed from the tank reveals the formation of spatial memory during training. Time spent in the training quadrant during the probe trial at the end of the first day of training was not different between groups and was at chance level (dotted line). By the last day of training there was clear spatial memory formation in sham-infected (SHAM) mice and in infected mice treated with ritonavir (TMEV+RIT), but vehicle-treated infected mice (TMEV+VEH) still performed at chance. *P *< 0.001 by one-way ANOVA; SNK pairwise analysis indicates *P *< 0.001 for ritonavir vs vehicle, *P *< 0.001 for sham vs vehicle, and *P* = 0.186 for sham vs vehicle. n = 20 mice per treatment in two separate experiments. (**b**) A discrimination index was calculated as the ratio of time spent investigating a novel object to the time spent investigating a familiar object. Sham-infected (SHAM) mice, infected mice treated with ritonavir (RIT), and infected mice treated with E64 showed indices above 2, consistent with the formation of novel object recognition memory. In contrast, infected (TMEV) mice and infected mice treated with vehicle (VEH) or PBS had indices close to 1, indicating equal time spent investigating the novel and the familiar object (dotted line). *P *< 0.001 by one-way ANOVA; SNK pairwise analysis indicates *P *< 0.001 for RIT vs VEH, *P* = 0.835 for RIT vs E64, *P* = 0.718 for SHAM vs RIT, *P* = 0.762 for E64 vs SHAM, *P *< 0.001 for E64 vs VEH. n = 10 mice per treatment in two separate experiments. Graphs show means ± 95% confidence intervals.

We have shown that destruction of CA1 neurons is correlated with loss of cognitive function in the Morris water maze and in a novel object recognition test^8^,^14^. We found that the ability to learn and recall the location of the hidden escape platform within the Morris water maze was lost in vehicle-treated mice when tested starting at 15 dpi but was completely preserved in animals treated with intraperitoneal ritonavir (treatment from time of infection through 4 dpi) (Fig. 4a). Indeed, performance in ritonavir-treated mice was at the same level as sham-infected mice (sham vs vehicle-treated, *P *< 0.001; sham vs ritonavir-treated, *P* = 0.186; ritonavir vs vehicle, *P *< 0.001). Likewise, the ability to discriminate the novel object was absent in TMEV-infected mice treated with vehicle but was preserved to sham-infected levels in ritonavir-treated mice (Fig. 4b) (sham vs TMEV, *P *< 0.001; sham vs TMEV + vehicle, *P *< 0.001; sham vs TMEV + ritonavir, *P* = 0.718; ritonavir vs vehicle, *P *< 0.001). Ritonavir-mediated preservation of cognitive function in the novel object recognition assay was equivalent to E64-mediated protection (Fig. 4b) (sham vs TMEV + E64, *P* = 0.762; E64 vs RIT, *P* = 0.835; E64 vs vehicle, *P *< 0.001), further linking calpain inhibition to neuroprotection in acutely infected mice.

### Therapeutic dosing with ritonavir protects the hippocampus and prevents seizures

**Figure 5:**
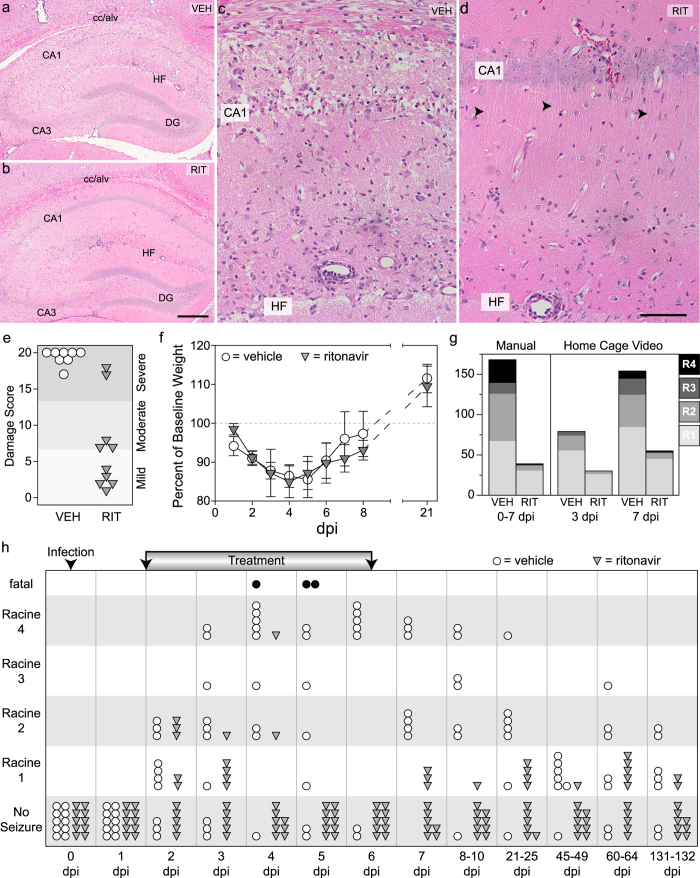
Ritonavir treatment initiated after disease onset protects the hippocampus and prevents escalation of behavioral seizures. Mice were treated with oral ritonavir (RIT) or vehicle (VEH) every 6 hrs starting at 36 hours postinfection. VEH mice showed extensive hippocampal injury at 7 dpi (**a**,**c**) but RIT mice were protected (**b**,**d**). Most of the CA1 neurons in RIT mice were intact (**b**) and maintained apical dendrites (arrows in **d**). Images are representative of 10 mice per group in two separate experiments. (**e**) Quantitation of hippocampal injury revealed that VEH mice had almost complete bilateral damage to CA1 (0 = no damage; 10 = 50% bilateral damage or 100% unilateral damage; 20 = 100% bilateral damage), while most RIT mice had only mild or moderate injury. *P *< 0.001 by t-test; n = 10 per group; representative of more than 40 mice in three separate experiments. (**f**) Body weights were not different between VEH (open circles) and RIT (filled inverted triangles) mice. *P* = 0.923 by two-way RM-ANOVA; n = 10 per group. (**g**) Behavioral seizures were quantified as described in the methods. The total number of Racine events manually observed between 0 and 7 dpi (*P *< 0.001 by Fisher exact test; n = 10 per group) and the total number of Racine events detected by video at 3 and 7 dpi (P = 0.136 at 3 dpi; P = 0.001 at 7 dpi by Fisher exact test; n = 10 per group in two experiments) were reduced in RIT mice. (**h**) Maximum Racine score observed each day in VEH (open circles) and RIT (filled inverted triangles) groups is shown. Both groups started with 10 mice; 3 VEH mice died (black circles) during the experiment; no RIT mice died. Ritonavir suppressed seizures from 3 dpi until the end of the experiment (132 dpi); *P *< 0.001 between treatments by two-way RM-ANOVA; day 4-132 significant at *P *< 0.001 by SNK pairwise analysis; 3 dpi significant at *P* = 0.023. Scale bar in (**b)** is 500 μm and refers to (**a**) Scale bar in (**d)** is 100 μm and refers to (**c)**. Graphs show means ± 95% confidence intervals.

Finally, because our ultimate goal is to identify therapeutically relevant strategies for protecting neurons during acute infection of the brain, we tested the neuroprotective efficacy of an oral ritonavir dosing regimen that started 36 hrs after infection. This timepoint was chosen for four reasons: 1) the peak inflammatory monocyte influx has already occurred^6^,^7^; 2) calpain levels have already started to increase in the hippocampus but have not peaked (Fig. 1e); 3) hippocampal neuron loss is already evident by 48 hpi^8^; and 4) behavioral seizures have already started but have not reached maximal levels (Fig. 5h). We delivered pharmaceutical grade ritonavir or vehicle by oral gavage every 6 hr starting at 36 hpi, continuing until 96 hpi, and then delivered one dose at 114 hpi and at 138 hpi. Mice were killed at 168 hpi for histological analysis. While vehicle-treated mice showed profound damage to CA1 (Fig. 5a,c), the majority of ritonavir-treated mice exhibited robust protection (Fig. 5b,d), despite the clear presence of ongoing inflammation (Fig. 5d). Indeed, 50% of the ritonavir-treated mice had mild hippocampal injury scores^14^ while only 20% of the ritonavir-treated group exhibited severe damage (Fig. 5e). All of the vehicle-treated mice had severe damage scores and the difference between ritonavir and vehicle was statistically signficant (*P *< 0.001 by t-test). Longitudinal assessment of body weight revealed no difference between vehicle and ritonavir groups with regard to systemic illness effects (Fig. 5f), suggesting that ritonavir did not simply stop or reverse infection and inflammation.

Therapeutic dosing with ritonavir almost completely reversed the development of severe seizures, as assessed via two different methods. In the first method, mice were moved to observation cages for 2 hr epochs of direct visual seizure scoring at the same time every day from 0 to 7 dpi. In the second method, mice were video recorded in their home cage for a 6 hr epoch during the same period each day at 3 and 7 dpi and the videos were scored offline for seizure behavior. Counting all manually observed Racine events over the first 7 days of infection revealed that ritonavir robustly suppressed moderate-to-severe seizures as compared to vehicle treatment (*P *< 0.001 by Fisher exact test) (Fig. 5g). Likewise, home cage recordings showed that ritonavir suppressed moderate-to-severe seizures at 7 dpi in the context of longer observation sessions and no direct manipulation of the mice (*P* = 0.136 at 3 dpi; *P* = 0.001 at 7 dpi by Fisher exact test) (Fig. 5g). Finally, analysis of the maximum daily Racine event observed for each individual animal revealed that ritonavir- and vehicle-treated mice were not statistically different with regard to seizures at 2 dpi, exhibiting the same pattern of low-to-moderate Racine scores (Fig. 5h). This is consistent with the fact that ritonavir treatment was only started 12 hr prior to this observation point. However, by 3 dpi, the ritonavir-treated group still showed low-to-moderate Racine events but the vehicle-treated group showed escalating seizure severity (Fig. 5h). At this point, the two groups were signficantly different (*P *< 0.001 between treatments at all timepoints by two-way repeated measures ANOVA; *P* = 0.023 at 3 dpi by SNK pairwise analysis). By 4 dpi, the protective effect of ritonavir was robust and the majority of treated mice showed no Racine seizure events (*P *< 0.001 by SNK pairwise analysis). Indeed, at 5 and 6 dpi the ritonavir cohort showed no Racine events while the vehicle-treated group showed severe seizures, including several mortalities. While the ritonavir cohort showed mild Racine events at later timepoints, out to 132 dpi, the group remained statistically different from the vehicle-treated group throughout the experiment (*P *< 0.001 at all remaining timepoints by SNK pairwise analysis). We conclude that treatment started even after the peak of inflammatory monocyte infiltration and the initiation of seizures is sufficient to preserve hippocampal circuitry.

### Ritonavir treatment does not alter monocyte infiltration

**Figure 6:**
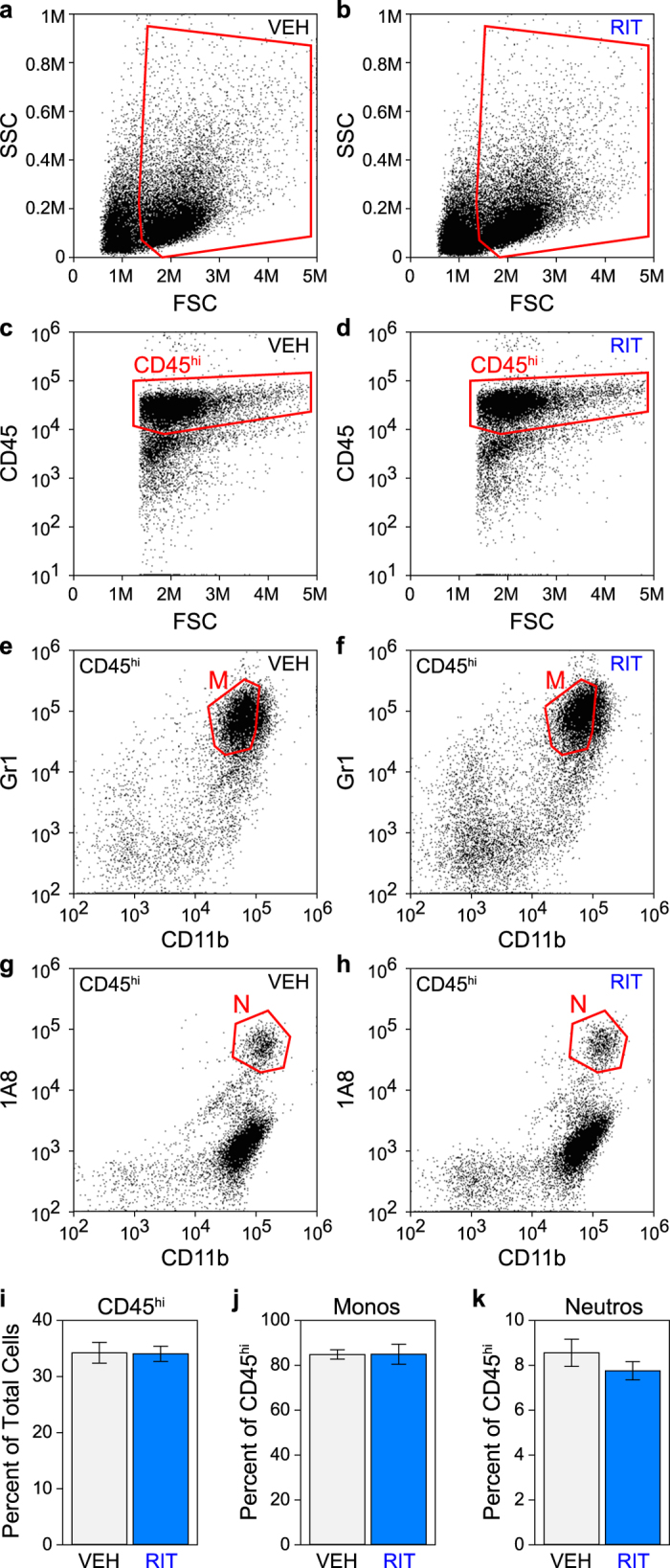
Ritonavir treatment does not alter monocyte infiltration. Mice were treated with oral ritonavir (RIT) (**b**,**d**,**f**,**h**) or vehicle (VEH) (**a**,**c**,**e**,**g**) at 36 hours postinfection and brain-infiltrating leukocytes were collected at 48 hours postinfection. The isolated cells did not differ by forward and side scatter profile (**a**,**b**) and the percent of CD45^hi^ cells (**c**,**d**,**i**) present in the isolate was not different between treatment groups (*P* = 0.865 by t-test). Cells in (**c** and **d)** are from gates shown in (**a** and **b)** respectively (outlined in red). The percentage of CD11b^+^Gr1^+^ monocytes (M, gate outlined in red in **e** and **f**) present in the CD45^hi^ population (gated in red in **c** and **d**) did not differ between groups (**j**) (*P* = 0.962 by t-test; n = 5 mice per group). The percentage of CD11b^+^1A8^+^ neutrophils (N, gate outlined in red in **g** and **h**) was statistically different between treatment groups (**k**) (*P* = 0.0159 by t-test; n = 5 mice per group) but was only a difference of less than 1%. Graphs show means ± 95% confidence intervals.

Our data suggest that hippocampal preservation and seizure suppression are the result of ritonavir-mediated inhibition of calpain activity in CA1 pyramidal neurons (Fig. 1). However, systemic delivery of ritonavir and subsequent inhibition of calpain could also interfere with infiltration of the inflammatory monocyte population responsible for triggering neuronal injury in the first place^6^,^7^. Though this is unlikely given the timing disparity between the initiation of the injury process (<24 hpi) and the initiation of ritonavir therapy (36 hpi), we measured the effects of treatment on brain-infiltrating leukocytes. Mice were treated with oral ritonavir or vehicle at 36 hpi and brain-infiltrating leukocytes were prepared at 48 hpi (Fig. 6). The isolated cells did not differ by forward and side scatter profile (Fig. 6a,b) and the percent of CD45^hi^ cells (Fig. 6c,d) present in the isolate was not different between groups (P = 0.865 by t-test) (Fig. 6i). The percentage of CD11b^+^Gr1^+^ monocytes (Fig. 6e,f) in the CD45^hi^ population also did not differ between groups (Fig. 6j) (P = 0.962 by t-test). The percentage of CD11b^+^1A8^+^ neutrophils (Fig. 6g,h) was statistically different between treatment groups (Fig. 6k) (P = 0.0159 by t-test), but this difference was less than 1% and neutrophils represented only a small percentage of the infiltrate at this timepoint. We conclude that ritonavir-mediated neuroprotection was not the result of alterations in monocyte infiltration, lending further support to the hypothesis that protection was mediated by inhibition of calpain in neurons.

## Discussion

Given the deep evolutionary roots of innate immune responses to tissue injury^19^,^20^,^21^,^22^, it is likely that neuroinflammation is associated with every pathogenic perturbation of neural homeostasis. Moreover, the diverse repertoire and complex and dynamic nature of innate immune cell functions makes it likely that the neuroinflammatory response participates simultaneously in pathogenic, protective, and reparative aspects of neurologic disease – the same infiltrating immune cell that is pathogenic at one timepoint may be critically protective at another. On this basis it is challenging to consider direct manipulation of immune cell infiltration or function as a therapeutic strategy for reducing CNS immunopathology, particularly within the context of viral encephalitis^23^. Therefore, rather than target the inflammatory cells directly, it becomes necessary to intervene at the level of the neural cells injured by the inflammatory response.

As we have previously shown in the TMEV model system using the Daniel’s strain and C57BL/6J mice, infected neurons are not killed by the virus, are not targeted by antiviral T cells, and clear virus in a non-destructive manner^7^,^8^, consistent with other neuroviral models^24^. Instead, the cell death cascade initiated in CA1 hippocampal neurons arises in response to the infiltration of inflammatory monocytes. As we have shown, blocking inflammatory monocytes results in extensive neuroprotection and maintenance of cognitive performance in acutely infected mice^6^,^7^. The release of proinflammatory cytokines such as IL-6^25^,^26^, IL-1β^27^, and TNFα, and the production of nitric oxide and other inflammatory mediators by infiltrating inflammatory monocytes^28^ likely creates an environment in the brain that triggers hyperexcitation within the hippocampal circuit^29^. As we have shown, inflammatory monocytes densely infiltrate the CA1 region within hours of infection in C57BL/6J mice^6^ and retraction of CA1 apical dendrites is observed shortly thereafter^8^. Apical dendrite retraction results in loss of inhibitory control of the CA3-to-CA1 circuit and is a hallmark of hyperexcitability^30^. In SJL mice infected with the Daniel’s strain of TMEV there is limited inflammatory monocyte infiltration^7^ and these animals do not exhibit hippocampal injury or retraction of apical dendrites^7^ and do not experience seizures^31^, further suggesting a link between inflammation and hyperexcitability in this model.

Neuronal excitotoxicity in CA1 is likely both a response to and a cause of further destabilization of the hippocampal circuit. While the mechanisms linking CA1 neuron cell death to seizure induction in our model are yet to be fully elucidated, it is likely that an early loss of feedforward connections from CA1 to the entorhinal cortex alters feedback inhibition of the hippocampal circuit in a manner that exacerbates and spreads the cell loss through CA1. Notably, we have previously shown that the earliest signs of CA1 pyramidal neuron dropout are observed at the CA2-CA1 border, spreading from proximal CA1 to distal CA1 as the acute pathology progresses^8^. Indeed, the damage to proximal CA1 is likely what underlies the loss of spatial memory function^32^. Recent evidence suggests that the medial entorhinal cortex, which projects to CA1 and elsewhere through the hippocampal axis, provides excitatory input on paravalbumin-positive basket cells that inhibit pyramidal neurons^33^. Thus, one possible model of seizure induction in our model is acute inflammation-induced hyperexcitability and death of a subset of proximal CA1 neurons that project to the subiculum, resulting in decreased excitation of medial entorhinal neurons that project back more widely onto CA1 inhibitory basket cells, resulting in decreased local inhibition and further CA1 pyramidal neuron hyperexcitability and cell death^34^,^35^.

The role of calpains in neuronal death during excitotoxicity and in response to seizures and circuit dysregulation is well established^36^,^37^,^38^. Furthermore, calpain inhibitors, including ritonavir, are well-documented as neuroprotective agents in both *in vitro* and *in vivo* models of excitotoxicity and neurotoxicity^13^,^39^. On this basis we favor a strong interpretation of our data: ritonavir-mediated inhibition of calpain activity protects CA1 pyramidal neurons from excitotoxicity- and inflammation-induced cell death^40^, short-circuiting the development of seizures and preserving cognitive function. However, it is possible that ritonavir acts indirectly or via an off-target mechanism to elicit the protective phenotype. For example, ritonavir could alter leukocyte interactions with the vasculature to change infiltration kinetics, either via effects on calpain or by altering proteasome function. Or it could block inflammatory monocyte trafficking^41^, activation, or effector function^42^. Several factors argue against these possibilities. First, we found no difference in viral clearance in ritonavir-treated mice, suggesting that the drug does not alter the immune-mediated mechanisms of viral control, such as CD8^+^ T cell infiltration. Second, we achieved robust hippocampal protection and seizure inhibition when ritonavir therapy was started at 36 hrs after infection. As we have previously published, inflammatory monocytes are already present in the brain at high levels by 12 hrs after infection and peak by 18–24 hrs^6^. Therefore, initiation of ritonavir at 36 hrs occurs too late to alter the peak inflammatory monocyte response. Moreover, analysis of brain-infiltrating leukocytes at 48 hrs after infection revealed no differences in CD45^hi^CD11b^+^Gr1^+^ monocytes in ritonavir-treated mice (Fig. 6). Third, several studies have shown that monocyte and neutrophil chemotaxis, survival, phagocytosis, and oxidative responses to pathogens are *increased* in HIV-infected patients treated with ritonavir^43^,^44^, and even cells from healthy controls exhibited enhanced migratory capacity in response to ritonavir^43^. Likewise, analysis of HIV-infected pediatric patients showed that ritonavir, as part of highly active antiretroviral therapy, did not reduce monocyte production of TNFα over the course of 24 weeks of treatment and, in fact, increased production of the stimulating cytokine IL-12p70^45^. Fourth, 96 weeks of ritonavir therapy (either in conjunction with atazanavir or darunavir) had no significant effect on the number of circulating CD14^+^CD16^+^ monocytes or CD14^dim^CD16^+^ non-classical monocytes^46^. Fifth, even direct *ex vivo* incubation of leukocytes with ritonavir, at concentrations similar to maximum one hour post-treatment plasma levels, only weakly impacted oxidative responses and had little to no effect on phagocytosis^47^. Sixth, direct *in vitro* incubation of monocytic cells with 1 μM ritonavir for 4 days did not reduce superoxide production and did not alter proliferative responses^48^. Finally, even if ritonavir therapy does alter some aspect of leukocyte infiltration or function that we have not assessed, the mice are still protected from hippocampal injury and seizures without jeopardizing viral clearance or health of the host, supporting our contention that ritonavir treatment may be a unique neuroprotective intervention during acute viral encephalitis^41^.

An important caveat regarding the neuroprotective effect of ritonavir in our model system is that this drug exhibits both inhibitory and activating interactions with other proteases and other cellular injury pathways. Indeed, given that ritonavir was originally developed as an HIV protease inhibitor, we have exploited the off-target effect of this drug on calpain activity for our study. It has been shown that ritonavir simultaneously inhibits the chymotrypsin-like activity of the proteasome while enhancing tryptic activity of this complex^49^,^50^ and sensitizes cells to other proteasome inhibitors via induction of ER stress and changes in histone acetylation^51^,^52^. Ritonavir also activates the unfolded protein response^53^,^54^, induces reactive oxygen species production and oxidative stress^55^, and triggers cellular senescence^56^, suggesting a panoply of possible mechanisms-of-action in our model system. However, given that ritonavir exerted a neuroprotective effect in our animals, the engagement of these alternative effector functions were either insufficient to cause or exacerbate injury or were themselves neuroprotective.

Our model is the first to show that inhibition of calpain protects neurons from inflammation-induced death during acute viral encephalitis. Our model is also the first to show that inhibition of calpain prevents the development of inflammation-induced seizures. The implication of our findings is that calpain inhibitor therapy, whether with ritonavir or other calpain inhibitors currently under development, such as BDA-410^16^,^57^,^58^, MDL-28170^34^,^59^, and SNJ-1945^41^,^60^,^61^,^62^, may be a reasonable strategy to limit neuropathology, seizures, and cognitive impairment associated with acute neuroinflammatory conditions triggered by infection. Notably, given the widespread use of ritonavir as a booster in highly active antiretroviral therapy^63^, this drug has likely been taken in excess of a billion times by humans. While ritonavir does have side effects associated with oral delivery, the incidence of severe complications is low^64^. Importantly, ritonavir is well-tolerated in pediatric patients^65^. Our pharmacokinetics data suggest that ritonavir may accumulate in the CNS within the context of acute inflammation, perhaps due to changes in permeability at the blood-brain barrier. Such accumulation may positively impact the efficacy of ritonavir therapy by concentrating the drug at sites of ongoing injury or more globally throughout the brain parenchyma. However, it is also possible that such accumulation could lead to complications in recovery of barrier function or trigger other negative sequelae such as neurotoxicity. Direct exposure of neurons in culture to various concentrations of ritonavir revealed that this drug had moderate to negligible toxicity at doses normally measured in CSF^66^, but accumulation within brain microdomains during infection could drive local concentrations into a toxic range. Likewise, ritonavir treatment has been shown to induce metabolic and cognitive changes in normal mice when delivered daily for 3 weeks^67^, suggesting that longer-term treatment may have unacceptable side effect profiles. Future studies testing the safety and tolerability of ritonavir during acute neuroinflammation may be required to rule out such negative effects. Nonetheless, in our animal model there were no apparent deleterious effects of short-term treatment with ritonavir during viral encephalitis, suggesting that this drug may serve as an acute therapeutic neuroprotective intervention.

Overall, our observations suggest that ritonavir may be a unique neuroprotective drug that could serve as a first line of defense in patients susceptible to inflammation-mediated seizures and neural injury, including adult and pediatric patients acutely infected with neurovirulent strains of enteroviruses (e.g. EV71), influenza viruses, paramyxoviruses (e.g. measles, mumps, Nipah), and flaviviruses (e.g. Dengue, West Nile, Zika, tick-borne encephalitis virus, Japanese encephalitis virus). Expansion of neurovirulent RNA viruses such as Zika virus into new geographic ranges as a result of global climate dyshomeostasis^68^ may mean that emergent and re-emergent CNS viral disease is on the rise^69^ and a concomitant increase in neural injury and cognitive sequelae induced by the acute inflammatory response to CNS infection may be an incipient socioeconomic problem^70^.

## Materials and Methods

### Mice and virus

C57BL/6J (#000664) mice were acquired from The Jackson Laboratories (Bar Harbor, ME). Females were used for all experiments. This study was approved by the Mayo Clinic IACUC and all experiments were performed in accordance with institutional and National Research Council guidelines^71^. At 6–8 weeks of age, mice were infected by intracranial injection of 2 × 10^5^ PFU of the Daniel’s (DA) strain of TMEV in 10 μL DMEM, prepared as previously described^72^. Sham-infected mice received intracranial injection of 10 μL virus-free DMEM. Injections were performed free-hand into the frontal cortex by an expert using the technique we and others have extensively employed for this model system^73^.

### Drug treatments

Norvir, the pharmaceutical preparation of ritonavir, consists of 80 mg/mL ritonavir, 43.2% (v/v) ethanol, polyoxyl 35 castor oil, propylene glycol, anhydrous citric acid, and peppermint oil. For oral administration, ritonavir was diluted in PBS (0.5 mg ritonavir per 1 g body weight) just prior to administration. A vehicle solution was prepared with 43% ethanol, 105 mg/mL Cremophor EL, 0.25 mg/mL propylene glycol, 3.5 mg/mL peppermint oil, and 2.8 mg/mL citric acid^74^. For intraperitoneal delivery and the *in vitro* analyses, powdered ritonavir (Abbott Laboratories) was dissolved in DMSO at 100 μg/mL and diluted in DMEM immediately prior to use. E64 was dissolved in PBS.

### Pharmacokinetics

Ritonavir plasma concentrations were measured by reverse phase liquid chromatography-tandem mass spectrometry using a Shimadzu liquid chromatograph coupled to a triple quadropole Quattro mass spectrometer. Quinoxaline was used as an internal standard. Plasma was collected in EDTA-coated tubes and proteins were precipitated with acetonitrile. A standard curve was prepared by spiking normal mouse plasma with ritonavir (1–1000 ng/mL). For analysis of hippocampal ritonavir levels, mice were perfused with PBS prior to tissue collection. The hippocampus was homogenized in ice-cold PBS and supernatant was collected after high-speed pelleting. The method was sensitive (1 ng/mL), linear over the concentration range (1–1000 ng/ml), and accurate (to within 15%).

### Calpain analyses

For the *in situ* calpain zymography, we followed the protocol of Duffy and Duffy^15^. Calpain activity in the hippocampus was assessed biochemically using the luminescent Calpain-Glo (Promega) cell-free cleavage assay.

### Brain-infiltrating leukocyte preparation and flow cytometry

Brain-infiltrating cells were prepared following our previously published protocol^73^. Cells were blocked with unconjugated anti-CD16/32 (clone 93; BioLegend), then surface labeled with PerCP-conjugated anti-CD45 (BD Pharmingen, clone 30-F11), FITC-conjugated anti-CD11b (BD Pharmingen, clone M1/70), and PE-conjugated anti-Ly6C/G (Gr1; BD Pharmingen, clone RB6-8C5) or anti-Ly6G (BD Pharmingen, clone 1A8) at 1:200 dilution for 30 min at 4 °C. Flow cytometric analysis was performed on an Accuri C6 and gates were applied as previously described^6^. Data were subsequently analyzed in FlowJo and SigmaPlot (Systat Software).

### Histology and Terminal Deoxynucleotidyl Transferase dUTP Nick-End Labeling (TUNEL)

Mice were perfused via intracardiac puncture with 50 mL of 4% paraformaldehyde and tissues were postfixed in 4% paraformaldehyde for 24 hours. The brain was macrosectioned by making cuts through the optic chiasm and the infundibulum and the resulting three brain sections were embedded in one block of paraffin, sectioned at five micron thickness, mounted on charged slides, rehydrated, and stained with H&E. For TUNEL staining, sections were microwave-treated in 0.1 M citrate buffer, washed once with PBS, and blocked at room temperature with 3% bovine serum albumin and 20% fetal calf serum in Tris buffer for 1 hour. Slides were rinsed three times with PBS, labeled with TUNEL reaction mix (Roche) for one hour at 37 °C, rinsed, and mounted in hard-set mounting media containing 4,6-diamidino-2-phenylindole (DAPI, Vector Laboratories). TUNEL-positive cells were counted using our previously established method^8^.

### Microscopy

Fluorescence and bright-field images were captured with an Olympus DP73 camera mounted on a Zeiss Axioskop 40 using Olympus Cellsens acquisition software. All objectives were Zeiss pan-Neofluar (5x = 0.15 N.A.; 10x = 0.30 N.A.; 20x = 0.50 N.A.). Images were collected at room temperature without refractive imaging medium. The *in situ* calpain zymography utilized TAMRA-conjugated casein (Anaspec); TUNEL was performed with fluorescein-dUTP (Roche). Image post-processing was performed in Image J and Photoshop. Levels were normalized, when appropriate, equally across images; gamma values were not changed.

### Behavioral tests and seizure analysis

We followed our previously published protocols for water maze and novel object recognition^8^,^14^. For direct manual scoring of behavioral seizures, all Racine^75^,^76^ scoreable events observed during a 2 hr epoch collected at the same time every day were measured, as previously described^77^. In these experiments, groups of 5 mice were moved from their home cage to a disposable plastic observation cage. A new cage was used for every scoring session for every group. For video analysis of seizures, mice in their home cage were video recorded for 6 hr epochs collected at the same time every day. Racine scoreable events were measured post hoc. For this study, we used a modified Racine scale: 1 = repetitive mouth and facial movements; 2 = head nodding and immobility; 3 = forelimb clonus; 4 = rearing and falling. Scores were generated by an expert reviewer blinded to the identity of the mice. Three scorers were used for some epochs to provide a check on the reproducibility of the primary expert. While the number of Racine 1 counts exhibited some variability between scorers, consistent with the subtlety of these events, the overall reproducibility between the expert and other scorers was P = 0.920 by Pearson chi-square analysis.

### LDH assay

Hippocampal neurons (prepared from embryonic day 18 mice) at 10 days *in vitro* were incubated for 24 hr with the calcium ionophore A23187 dissolved in DMSO. Maximal release samples were generated by lysis with 2% TX-100. Samples of supernatant were incubated with reaction solution (7.2 mg/mL sodium L-lactate, 0.4 mg/mL iodonitrotetrazolium chloride, 0.03% BSA, 1.2% sucrose, 1350 mU diaphorase, 300 μg NAD+, in PBS) for 1 hr at RT. LDH release was determined by subtracting the absorbance at 650 nm from the absorbance at 510 nm and calculating the relative ratio to maximal release.

### Plaque assay

L2 cells were plated at 5 × 10^5^ cells per well in 12-well plates two days prior to assay. For measurement of viral load *in vivo*, the brain was weighed and homogenized with a rotor-stator homogenizer on ice in 10 volumes (wt:vol) sterile PBS prepared with DEPC-treated water. Following clarification of the homogenate at 1000 g for 20 min at 4 °C, serial 10-fold dilutions were prepared and titered on L2 cells. Virus titer and the *in vitro* effect of calpain inhibitors on TMEV replication and fitness were assessed by incubating confluent L2 cells for 1 hr at 37 °C in 200 μL serum-free DMEM containing diluted brain homogenate or defined numbers of TMEV plaque-forming units (PFU). After inoculation, cells were overlaid with 1 mL 1% SeaPlaque agarose prepared in DMEM. After 48 hr, cells were fixed with EtOH:HOAc:formaldehyde (6:2:1 v-v:v) for one hr, then rinsed in water and stained with 1% crystal violet prepared in 20% EtOH. Plaques were counted in triplicate inoculates.

### Statistics

α = 0.05 and β = 0.2 were established a priori. Post hoc power analysis was performed for all experiments and significance was only considered when power ≥0.8. Statistical analysis was performed using SigmaPlot (Systat Software). Normality was determined by the Shapiro–Wilk test and normally distributed data were checked for equal variance. Parametric tests were only applied to data that were both normally distributed and of equal variance. The Student–Newman–Keuls pairwise comparison test was used for all post hoc sequential comparisons following ANOVA. Curran-Everett guidelines were followed^78^. Error bars in all graphs are 95% confidence intervals.

## Additional Information

**How to cite this article**: Howe, C. L. *et al*. Neuroprotection mediated by inhibition of calpain during acute viral encephalitis. *Sci. Rep.* **6**, 28699; doi: 10.1038/srep28699 (2016).

## Supplementary Material

Supplementary Information
