# Supplementary material for: Neuroprotection mediated by inhibition of calpain during acute viral encephalitis
Source: Sci Rep. 2016 Jun 27;6:28699. doi: 10.1038/srep28699 (PMC4921808; doi:10.1038/srep28699)
Supplement: Supplementary Information [file srep28699-s1.pdf]

Title: Neuroprotection mediated by inhibition of calpain during acute viral encephalitis

Authors: Charles L Howe[1,2,3,*], Reghann G LaFrance-Corey[1], Kanish Mirchia[1], Brian M Sauer[4], Renee M McGovern[5], Joel M Reid[5], Eric J Buenz[1]

Affiliations:
Departments of [1]Neurology, [2]Neuroscience, and [3]Immunology, Mayo Clinic, Rochester, Minnesota, USA, 55905.
[4]Neurobiology of Disease PhD program, Mayo Graduate School, Mayo Clinic, Rochester, Minnesota, USA, 55905.
[5]Division of Oncology Research, Department of Oncology, Mayo Clinic, Rochester, Minnesota, USA, 55905.

*Correspondence to: Charles Howe, PhD, Guggenheim 1542C, Mayo Clinic, 200 First Street SW, Rochester, MN 55905; 507-284-9288; howe@mayo.edu

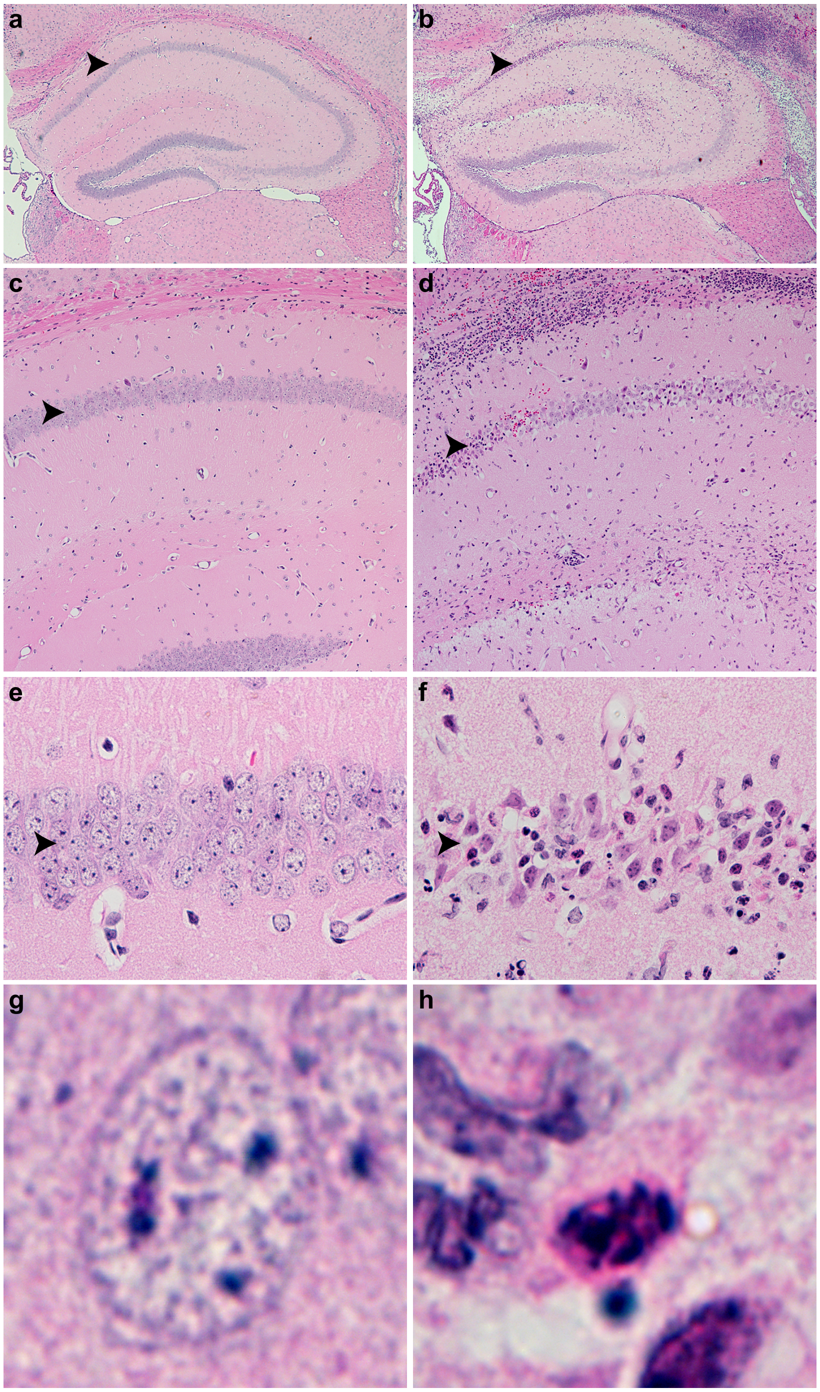

Supplemental Figure 1. Acute TMEV infection-induced neuropathology. Mice were either sham infected (a, c, e, g) or were inoculated with TMEV (b, d, f, h). At 3 dpi mice were killed, perfused with 4% PFA, and brains were processed for paraffin embedding and sectioning at 5 microns. H&E staining reveals robust injury to the CA1 layer (arrowheads) in TMEV-infected (b, d, f) but not sham-infected (a, c, e) mice. A healthy pyramidal neuron in a sham animal (g) compared to several pyknotic neurons in a TMEV animal (h) highlights the neuronal death phenotype.
